# Efficacy of Ustekinumab for the treatment of perianal fistulizing Crohn’s disease: a multicentre retrospective study

**DOI:** 10.3389/fmed.2026.1839761

**Published:** 2026-07-10

**Authors:** Youran Li, Xiaomei Sun, Ke Wen, Lu Lu, Wei Wang, Minna Wu, Jiaze Ma, Zailong Zhou, Yunfei Gu

**Affiliations:** 1 Department of Colorectal Surgery, Jiangsu Province Hospital of Chinese Medicine, Affiliated Hospital of Nanjing University of Chinese Medicine, Nanjing, Jiangsu, China; 2 Department of Colorectal Surgery, The Affiliated Hospital of Qingdao University, Qingdao, Shandong, China; 3 Department of Colorectal Surgery, Suzhou TCM Hospital Affiliated to Nanjing University of Chinese Medicine, Suzhou, Jiangsu, China; 4 Department of Gastroenterology, Shaoxing Hospital of Traditional Chinese Medicine, Shaoxing TCM Hospital Affiliated to Zhejiang Chinese Medical University, Shaoxing, Zhejiang, China

**Keywords:** biologic therapy, fistula healing, perianal fistulizing Crohn’s disease, predictive nomogram, Ustekinumab

## Abstract

**Background:**

Perianal fistulizing Crohn’s disease (pfCD) is a refractory complication of Crohn’s disease (CD) with limited therapeutic options, particularly for patients failing anti-tumor necrosis factor agents. Ustekinumab (UST) shows promise in pfCD, but long-term outcomes and predictive factors remain unclear.

**Methods:**

This multicenter retrospective study enrolled 163 pfCD patients treated with UST (2020–2023) with a median 15-month follow-up. Primary endpoints were fistula response and clinical remission rates. Univariate/multivariate logistic regression identified healing predictors, and a nomogram was developed for outcome prediction.

**Results:**

At final follow-up, the fistula response rate was 88.3% and clinical remission rate 70.1%. Significant improvements were observed in inflammatory biomarkers (CRP: 21.2 ± 28.3 → 5.2 ± 8.9 mg/L; ESR: 36.6 ± 24.5 → 14.5 ± 18.3 mm/h) and hemoglobin (126.8 ± 18.5 → 148.2 ± 25.6 g/L; all *p* < 0.001). Definitive surgery (adjusted OR = 9.613, *p* < 0.001) and fewer prior biologics (adjusted OR = 0.541, *p* = 0.006) were independent healing predictors. The nomogram exhibited good discriminative ability (AUC = 0.852 for 24-week remission). UST was well-tolerated (adverse event rate = 7.4%) with no severe safety issues.

**Conclusion:**

UST demonstrated clinically meaningful efficacy within a combined multidisciplinary strategy for pfCD, with fistula response and clinical remission rates of 88.3 and 70.1%, respectively, though MRI-confirmed healing was modest (14.2%). Definitive surgery and fewer prior biologic exposures predicted better fistula healing. The developed nomogram facilitates individualized treatment decision-making.

## 1 Introduction

Crohn’s disease (CD) is a chronic, relapsing inflammatory bowel disease characterized by transmural inflammation of the gastrointestinal tract. Perianal fistulizing Crohn’s disease (pfCD) is a common and severe complication, affecting 18.7–50% of CD patients during the disease course (1–3). PfCD is associated with significant pain, fecal incontinence, repeated infections, and reduced quality of life, posing substantial challenges to clinical management (4).

The management of pfCD requires a multidisciplinary approach combining medical therapy, surgical intervention, and supportive care (5). Biologic agents have revolutionized the treatment of pfCD, with anti-tumor necrosis factor (anti-TNF) agents being the first-line biologic therapy (6). However, 10–40% of patients fail to respond to anti-TNF therapy, and secondary loss of response is common (7). For these patients, alternative biologics with distinct mechanisms of action are needed.

Ustekinumab (UST), a fully human monoclonal antibody targeting the p40 subunit of interleukin-12 and interleukin-23, has been approved for the treatment of moderate-to-severe luminal CD (6). Emerging evidence suggests that UST may also be effective in pfCD, but data on long-term outcomes and predictive factors for fistula healing remain inconsistent (8). Previous studies have reported remission rate (17.7 at week 24) with UST, and the impact of clinical factors such as prior biologic exposure, surgical intervention, and fistula complexity on treatment outcomes is not fully elucidated (9, 10). Current guidelines advocate for simultaneous multidisciplinary care combining medical therapy and surgical intervention, rather than sequential approaches. This study reflects real-world practice where surgery and biologics are integrated throughout treatment.

Furthermore, the lack of validated predictive tools for pfCD makes it difficult to individualize treatment strategies. Identifying patients who are likely to benefit from UST or require combined surgical intervention could optimize treatment outcomes and reduce healthcare burden. Therefore, this study aimed to evaluate the long-term efficacy and safety of UST in a large cohort of pfCD patients; identify independent predictors of fistula healing; and develop a clinical nomogram for individualized outcome prediction.

## 2 Method

### 2.1 Study design and population

This multicenter retrospective cohort study was conducted across seven tertiary referral hospitals in China: Jiangsu Provincial Hospital of Traditional Chinese Medicine (coordinating center), Longhua Hospital affiliated to Shanghai University of Traditional Chinese Medicine, Affiliated Hospital of Zhejiang Chinese Medical University, First Affiliated Hospital of Anhui University of Chinese Medicine, Beijing Anorectal Hospital, Suzhou Hospital of Traditional Chinese Medicine, and Anorectal Department of the Ninth People’s Hospital affiliated to Shanghai Jiao Tong University. This study was conducted across tertiary surgical referral centers specializing in perianal CD. The cohort is enriched with complex fistulas (89%) and prior surgical interventions (85.9%), reflecting the patient population typically referred for multidisciplinary surgical-gastroenterological management.

Consecutive patients with pfCD initiating UST between January 2020 and December 2023 were screened for inclusion. Data were collected through electronic medical records, outpatient visits, telephone interviews, and multimedia-based follow-up systems. The study was approved by the Ethics Committee of the Affiliated Hospital of Nanjing University of Chinese Medicine (2023NL-062-01) and formally approved at all participating centers. Written informed consent was obtained from all patients for retrospective data analysis.

Inclusion criteria were: (1) age 18–60 years; (2) established diagnosis of pfCD based on clinical, endoscopic, radiological, and/or histological criteria (11); (3) active draining perianal fistula at baseline; (4) initiation of UST therapy with intent-to-treat; and (5) minimum follow-up duration of 3 months. Exclusion criteria were: (1) active infection, intestinal obstruction, malignant tumor, or intestinal perforation during UST therapy; (2) intestinal resection or major abdominal surgery within 3 months before or during UST therapy; (3) severe cardiac, pulmonary, hepatic, or renal dysfunction; (4) insufficient follow-up or loss to follow-up before 3 months; and (5) incomplete medical records precluding outcome assessment.

### 2.2 Treatment protocol

All patients underwent pelvic MRI and clinical examination at baseline. Active abscesses were drained, and setons were placed for complex fistulas as clinically indicated. Asymptomatic fistulas or those with MRI diameter <1 cm without high-signal inflammation did not require drainage.

Intravenous Ustekinumab induction was administered at 6 mg/kg (260 mg, 390 mg, or 520 mg based on weight) at weeks 0 and 8, followed by subcutaneous maintenance (90 mg every 8 weeks). Dose escalation to every-4-week dosing was permitted for suboptimal responders after week 16, per physician discretion.

Fistula response was assessed at week 8 (first maintenance dose) and week 16. Patients with adequate response continued standard therapy; setons were removed if drainage ceased and no abscess was evident on MRI. Definitive surgical intervention (fistulotomy, advancement flap, or ligation of intersphincteric tract) was performed after endoscopic re-evaluation when technically feasible. Temporal sequencing: All patients received UST induction (Weeks 0 and 8) prior to definitive surgery, which was typically performed between Weeks 8–24 after initial inflammatory control. For suboptimal responders at week 16, intravenous re-induction with every-4-week dosing was implemented. New abscesses or fistula expansion prompted repeat drainage. Primary non-response led to transition to alternative biologics.

Fistula response was assessed at each maintenance visit. Final evaluation at week 52 (1 month after sixth maintenance dose) determined clinical outcomes. Patients with healed fistulas continued UST maintenance with periodic MRI surveillance.

### 2.3 Data collection and outcome definitions

Baseline demographic and clinical data were collected from electronic medical records, including age, gender, body mass index (BMI), smoking status, disease duration, disease location (L1–L4 according to the Montreal classification), disease behavior (B1–B3 + P), fistula classification (simple vs. complex according to the American Gastroenterological Association [AGA] criteria) (6), prior medical therapy (5-aminosalicylic acid, corticosteroids, immunosuppressants, biologics), and prior surgical history (perianal or abdominal surgery).

Follow-up data included inflammatory biomarkers (C-reactive protein [CRP], erythrocyte sedimentation rate [ESR], hemoglobin), disease activity indices (Crohn’s Disease Activity Index [CDAI], Perianal Disease Activity Index [PDAI], Wexner score), fistula outcomes (response, healing, recurrence), and adverse events.

The primary endpoint was fistula response, defined as a reduction of at least 50% in the number of draining fistulas compared to baseline (12). Clinical remission was defined as the absence of drainage from all external fistula openings upon gentle digital compression, consistent with standard definitions in pfCD trials (12, 13). Fistula healing rate on magnetic resonance imaging (MRI) is characterized by either the complete disappearance of previously detected high - signal tracts on MRI or the presence of residual tracts that have intermediate signal intensity along with low-signal boundaries. This reduction must be maintained over a minimum of two consecutive follow-up assessments. Given the limitations of clinical examination in detecting persistent subclinical disease activity, MRI healing was assessed as a co-primary endpoint alongside clinical remission. Outcomes were assessed at prespecified timepoints (weeks 8, 16, 24, 48, and final follow-up). Due to the retrospective design with enrollment spanning January 2020 to December 2023 and data cutoff in December 2023, patients enrolled after January 2022 had not completed 48 weeks of follow-up at the time of analysis. Consequently, the week 48 analysis represents a subset of patients with sufficient follow-up duration (*n* = 75), not a missing-data problem. The final follow-up analysis (median 15 months, IQR 9–22) includes all 163 patients at their actual last assessment.

### 2.4 Statistical analysis

Sample size was estimated based on anticipated fistula healing rate of 30% with UST, requiring 150 patients for 80% power to detect a 10% difference from historical controls (20% healing with conventional therapy), assuming 15% dropout.

Continuous variables were expressed as mean ± standard deviation (normal distribution) or median with interquartile range [IQR] (non-normal distribution). Categorical variables were presented as frequencies and percentages. Comparisons between timepoints utilized paired *t*-tests or Wilcoxon signed-rank tests for continuous variables, and Chi-squared or McNemar tests for categorical variables.

Univariate logistic regression identified factors associated with fistula response and healing. Variables with *p* < 0.10 in univariate analysis were entered into multivariate logistic regression models using backward elimination based on the Akaike information criterion. Odds ratios (OR) with 95% confidence intervals (CI) were calculated. A two-sided *p* < 0.05 was considered statistically significant. To address heterogeneity introduced by varying biologic exposure histories, all efficacy outcomes were stratified by biologic-naïve versus biologic-experienced status. Prior biologic exposure (dichotomized as yes/no and analyzed as number of prior biologics) was included as a covariate in both univariate and multivariate logistic regression models. A logistic regression-based nomogram was developed incorporating independent predictors of fistula healing. The events-per-variable (EPV) ratio was 28.5 (114 healing events / 4 predictors). Internal validation was performed using bootstrap resampling (1,000 iterations) to estimate optimism-corrected performance. Calibration was assessed using the Hosmer-Lemeshow test and visual calibration plots.

Statistical analyses were performed using SPSS version 26.0 (IBM Corp., Armonk, NY) and GraphPad Prism version 9.5.0 (GraphPad Software, San Diego, CA).

## 3 Results

### 3.1 Patient characteristics

**Figure 1:**
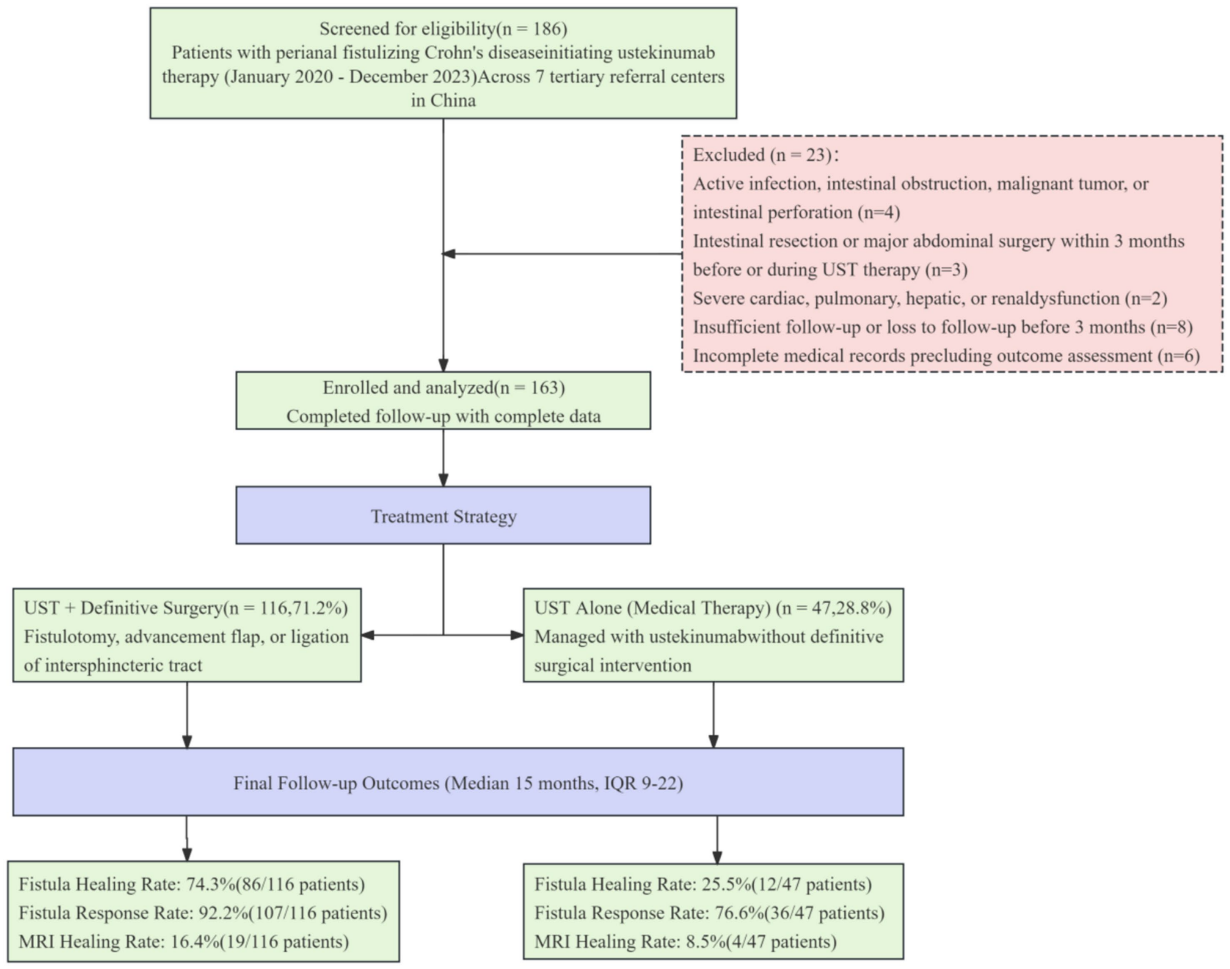
Flow diagram of patient selection and treatment allocation.

A total of 186 patients with pfCD initiating UST therapy between January 2020 and December 2023 were screened for inclusion across seven tertiary referral centers. Of these, 23 patients were excluded and 163 patients were enrolled with complete follow-up data (Figure 1). The baseline demographic and clinical characteristics are summarized in Table 1.

**Table 1:** Baseline characteristics of patients with perianal fistulizing Crohn’s disease treated with Ustekinumab.

Characteristic	Value
Demographics	
Total patients, *n*	163
Male sex, *n* (%)	120 (73.6)
Age at disease onset, median (IQR), years	26 (21–31)
BMI, mean ± SD, kg/m^2^	20.6 ± 3.2
Smoking, *n* (%)	1 (0.6)
Disease characteristics	
Disease duration, median (IQR), months	29 (15–64)
Perianal disease duration, median (IQR), months	35 (22–72)
Disease location, *n* (%)	
L1 (Ileal)	27 (16.6)
L2 (Colonic)	31 (19.0)
L3 (Ileocolonic)	98 (60.1)
L4 (Upper GI)	7 (4.3)
Disease behavior, *n* (%)	
B1 (Non-stricturing, non-penetrating)	45 (27.6)
B2 (Stricturing)	38 (23.3)
B3 (Penetrating)	80 (49.1)
Perianal disease modifier (P)	163 (100)
AGA fistula classification, *n* (%)	
Simple	18 (11.0)
Complex	145 (89.0)
Prior medical therapy, *n* (%)	
5-Aminosalicylic acid	93 (57.1)
Corticosteroids	21 (12.9)
Immunosuppressants	38 (23.3)
Anti-TNF agents	80 (49.1)
Infliximab	79 (48.5)
Adalimumab	6 (3.7)
Vedolizumab	6 (3.7)
Biologic-naïve	83 (50.9)
Prior surgical history, *n* (%)	
Perianal surgery	140 (85.9)
Abdominal surgery	10 (6.1)
Index treatment characteristics	
Definitive surgery during UST therapy	116 (71.2)
Number of setons, median (IQR)	1 (1–2)
UST dose escalation to every 4 weeks	23 (14.1)
Follow-up duration, median (IQR), months	15 (9–22)

The cohort was predominantly male (*n* = 120, 73.6%), with a median age at disease onset of 26 years (IQR, 21–31). The median body mass index (BMI) was 20.6 ± 3.2 kg/m^2^. The median disease duration of Crohn’s disease was 29 months (IQR, 15–64), while the median perianal disease duration was 35 months (IQR, 22–72). According to the AGA classification, 18 patients (11.0%) had simple fistulas, and 145 patients (89.0%) had complex fistulas. Regarding disease location, 27 patients (16.6%) had ileal involvement (L1), 31 (19.0%) had colonic involvement (L2), 98 (60.1%) had ileocolonic involvement (L3), and 7 (4.3%) had upper gastrointestinal involvement (L4). A substantial proportion of patients had prior surgical interventions: 140 patients (85.9%) had undergone previous perianal surgery, and 10 patients (6.1%) had a history of abdominal surgery. Definitive surgical intervention (fistulotomy, advancement flap, or ligation of intersphincteric tract) was performed in 116 patients (71.2%) during the UST treatment period. Prior medical therapies included 5-aminosalicylic acid in 93 patients (57.1%), corticosteroids in 21 (12.9%), immunosuppressants in 38 (23.3%), anti-TNF agents in 80 (49.1%), and vedolizumab in 6 (3.7%). Notably, 83 patients (50.9%) were biologic-naïve, while 80 patients (49.1%) had prior biologic exposure. The median number of setons placed was 1 (IQR, 1–2). The median follow-up duration was 15 months (IQR, 9–22 months). All patients received intravenous UST induction (6 mg/kg at weeks 0 and 8), followed by subcutaneous maintenance (90 mg every 8 weeks). Dose escalation to every-4-week dosing was implemented in 23 patients (14.1%) due to suboptimal response.

### 3.2 Efficacy of UST on luminal Crohn’s disease

**Figure 2:**
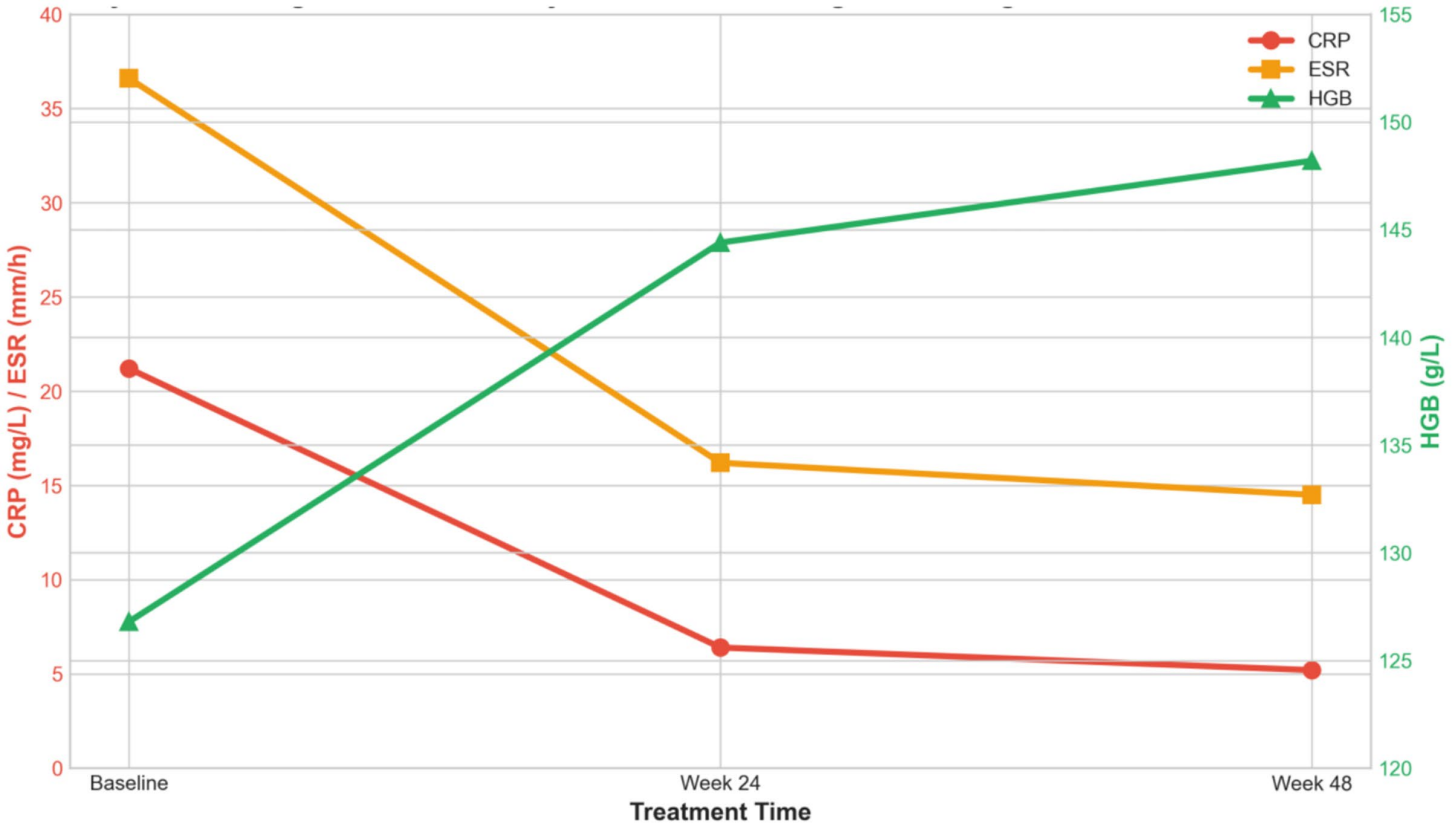
Dynamic changes of inflammatory markers and hemoglobin during Ustekinumab treatment. Data are presented as mean ± standard deviation. Numbers of patients with available data at each timepoint: baseline (*n* = 163), week 8 (*n* = 158), week 16 (*n* = 155), week 24 (*n* = 162), week 48 (*n* = 75), final follow-up (*n* = 163). Wide standard deviations reflect inter-individual variability in inflammatory response.

Following UST initiation, patients demonstrated significant improvements in systemic inflammatory markers and disease activity indices (Table 2). Dynamic changes in inflammatory and nutritional biomarkers are illustrated in Figure 2, which shows sustained reductions in CRP and ESR alongside progressive elevation of hemoglobin levels throughout the treatment course. CRP levels decreased significantly from baseline (median 21.2 mg/L, IQR 8.5–45.3) to week 24 (median 6.4 mg/L, IQR 2.8–15.2; *p* < 0.001). Notably, standard deviations were wide at all timepoints (e.g., CRP baseline SD 28.3 mg/L, week 24 SD 12.8 mg/L), reflecting substantial inter-individual variability in inflammatory trajectories. Similarly, ESR declined from median 36.6 mm/h (IQR 18.2–62.4) to 16.2 mm/h (IQR 8.5–28.7; *p* < 0.001) at week 24. Hemoglobin levels improved significantly from baseline (126.8 ± 18.5 g/L) to week 24 (144.4 ± 28.3 g/L; *p* = 0.001), indicating enhanced nutritional status. CRP normalization (CRP < 5 mg/L) was achieved in 55.6% of patients at week 24. Clinical remission, defined as CDAI <150, was observed in 82.1% of patients at week 24. The median CDAI decreased from 119.6 (IQR 82.1–170.2) at baseline to 41.1 (IQR 15.1–79.8) at week 24 (*p* < 0.001). Perianal Disease Activity Index (PDAI) scores also improved significantly from median 6.0 (IQR 4.0–9.0) at baseline to 1.8 (IQR 1.0–3.0) at week 48 (*p* < 0.001).

**Table 2:** Changes in inflammatory markers and disease activity indices following Ustekinumab initiation.

Variable	Baseline	Week 24	Week 48	*p*-value^*^
CRP, mg/L	21.2 ± 28.3	6.4 ± 12.8	5.2 ± 8.9	<0.001
ESR, mm/h	36.6 ± 24.5	16.2 ± 22.0	14.5 ± 18.3	<0.001
Hemoglobin, g/L	126.8 ± 18.5	144.4 ± 28.3	148.2 ± 25.6	0.001
Platelet count, ×10^9^/L	316.1 ± 92.5	278.0 ± 87.8	268.1 ± 108.8	0.396
CDAI	119.6 (82.1–170.2)	41.1 (15.1–79.8)	36.4 (12.8–68.2)	<0.001
PDAI	6.0 (4.0–9.0)	3.0 (2.4–4.0)	1.8 (1.0–3.0)	<0.001
Wexner score	7.0 (5.0–10.0)	4.0 (3.0–6.0)	3.0 (2.0–5.0)	<0.001

^*^p-values calculated using paired *t*-test for normally distributed continuous variables or Wilcoxon signed-rank test for non-normally distributed variables. Values presented as mean ± SD for normally distributed variables and median (IQR) for non-normally distributed variables.

### 3.3 Efficacy of UST on perianal fistulas

**Figure 3:**
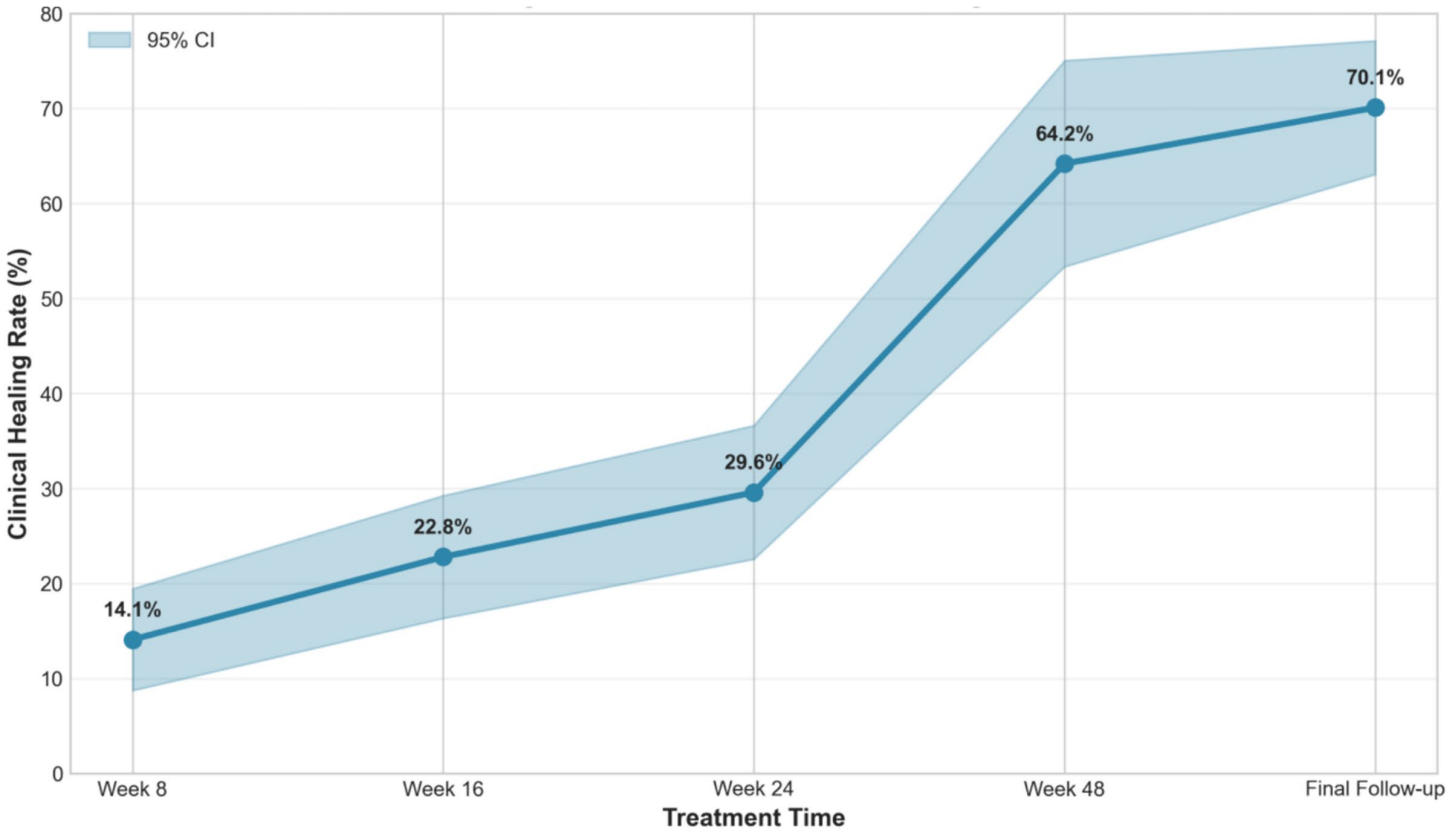
Trends in clinical remission rate of Perianal Fistulas During Ustekinumab treatment. The sharp increase between week 24 and week 48 reflects the timing of definitive surgical interventions (performed in 71.2% of patients during UST therapy), not purely pharmacological response.

The fistula response rate was 60.1% at week 8, increased to 74.7% at week 16, reached 88.7% at week 24, and was sustained at 88.3% at final follow-up (median 15 months). Week 48 assessment was available for 75 patients enrolled before January 2022; the fistula response rate in this subset was 46.7% (35/75). This lower apparent rate reflects right censoring of patients enrolled after January 2022 who had not reached 48 weeks at data cutoff, not differential attrition. The final follow-up response rate of 88.3% (143/163) at median 15 months represents the complete cohort (Table 3). The clinical remission rate increased from 14.1% at week 8 to 22.8% at week 16, 29.6% at week 24, 64.2% at week 48, and 70.1% at final follow-up (Figure 2). The sharp increase between week 24 and week 48 largely reflects the timing of definitive surgical interventions (71.2% of patients underwent surgery during UST therapy), rather than purely pharmacological response (Figure 3). MRI healing rates were 2.5% at week 16, 7.3% at week 24, and 14.2% at final follow-up. However, MRI improvement (including both complete healing and partial response) was observed in 30.8% of patients at week 24.

**Table 3:** Fistula response, clinical remission, and MRI healing rates at different timepoints.

Outcome	Week 8	Week 16	Week 24	Week 48	Final follow-up
Fistula response					
Response rate, *n* (%)	97 (60.1)	121 (74.7)	143 (88.7)	75 (46.3)	143 (88.3)
Clinical remission					
n with available data	162	162	162	75	163
Healing rate, n (%)	23 (14.1)	37 (22.8)	48 (29.6)	104 (64.2)	114 (70.1)
MRI healing					
*n* with available data	162	162	162	75	163
Complete healing, *n* (%)	—	4 (2.5)	12 (7.3)	5 (3.1)	23 (14.2)
Partial response, *n* (%)	—	18 (11.1)	38 (23.5)	28 (17.3)	45 (27.6)

Week 48 analysis includes only patients enrolled before January 2022 with ≥48 weeks of follow-up at data cutoff (December 2023). Lower apparent response rate reflects right censoring of later enrollees, not attrition bias. Final follow-up includes all patients at median 15 months (IQR 9–22).

### 3.4 Risk factors associated with fistula healing

Univariate analysis was performed to identify factors associated with fistula response and healing (Table 4). For fistula response, statistically significant factors included: median disease duration of CD (OR 0.987, 95% CI 0.978–0.995, *p* = 0.002), median perianal disease duration (OR 0.986, 95% CI 0.977–0.996, *p* = 0.004), history of abdominal surgery (OR 0.164, 95% CI 0.042–0.648, *p* = 0.010), immunosuppressant use (OR 0.171, 95% CI 0.063–0.465, *p* = 0.001), anti-TNF therapy (OR 0.148, 95% CI 0.041–0.529, *p* = 0.003), number of prior biologics (OR 0.410, 95% CI 0.222–0.758, *p* = 0.004), and number of setons placed (OR 0.406, 95% CI 0.230–0.717, *p* = 0.002). For fistula healing, significant predictors on univariate analysis included: prior anti-TNF therapy (OR 0.343, 95% CI 0.175–0.669, *p* = 0.002), number of prior biologics (OR 0.541, 95% CI 0.350–0.836, *p* = 0.006), and definitive surgery (OR 8.615, 95% CI 3.957–18.759, *p* < 0.001). Notably, gender, age at onset, BMI, disease duration, history of perianal or abdominal surgery, steroid use, immunosuppressant use, vedolizumab use, AGA classification, and number of setons were not significantly associated with fistula healing on univariate analysis.

**Table 4:** Univariate and multivariate logistic regression analyses of factors associated with fistula response and healing.

Variable	Fistula Response (Univariate) OR (95% CI)	*p*-value	Fistula Healing (Univariate) OR (95% CI)	*p*-value	Fistula Healing (Multivariate) Adjusted OR (95% CI)	*p*-value
Male sex (vs. female)	0.892 (0.452–1.761)	0.743	1.125 (0.587–2.158)	0.721	—	—
Age at onset (per year increase)	0.991 (0.968–1.014)	0.437	0.989 (0.966–1.013)	0.372	—	—
BMI (per kg/m^2^ increase)	1.012 (0.954–1.073)	0.698	1.008 (0.950–1.070)	0.764	—	—
CD disease duration (per month increase)	0.987 (0.978–0.995)	0.002	0.992 (0.983–1.001)	0.078	—	—
Perianal disease duration (per month increase)	0.986 (0.977–0.996)	0.004	0.991 (0.982–1.000)	0.053	—	—
Complex fistula (vs. simple)	0.785 (0.321–1.920)	0.596	0.894 (0.365–2.190)	0.807	—	—
Prior perianal surgery (vs. no)	0.876 (0.412–1.863)	0.732	1.052 (0.493–2.245)	0.896	—	—
Prior abdominal surgery (vs. no)	0.164 (0.042–0.648)	0.01	0.789 (0.245–2.538)	0.692	—	—
5-ASA use (vs. no)	1.123 (0.589–2.142)	0.723	1.087 (0.568–2.081)	0.801	—	—
Corticosteroid use (vs. no)	0.821 (0.356–1.892)	0.641	0.912 (0.394–2.110)	0.832	—	—
Immunosuppressant use (vs. no)	0.171 (0.063–0.465)	0.001	0.876 (0.389–1.972)	0.748	—	—
Anti-TNF exposure (vs. no)	0.148 (0.041–0.529)	0.003	0.343 (0.175–0.669)	0.002	0.342 (0.174–0.668)	0.002
Vedolizumab use (vs. no)	0.765 (0.213–2.748)	0.682	0.891 (0.247–3.210)	0.863	—	—
Number of prior biologics (per increase)	0.410 (0.222–0.758)	0.004	0.541 (0.350–0.836)	0.006	0.541 (0.350–0.836)	0.006
Number of setons (per increase)	0.406 (0.230–0.717)	0.002	0.872 (0.489–1.553)	0.642	0.515 (0.275–0.964)*	0.038*
Definitive surgery (vs. no)	1.234 (0.652–2.337)	0.521	8.615 (3.957–18.759)	<0.001	9.613 (4.047–20.747)	<0.001

*For fistula response multivariate model; other multivariate values are for fistula healing. *p-values <0.05 are considered statistically significant.

Multivariate logistic regression analysis identified definitive surgery as the strongest independent predictor of fistula healing (adjusted OR 9.613, 95% CI 4.047–20.747, *p* < 0.001). Each additional prior biologic agent reduced the odds of healing by 46% (adjusted OR 0.541, 95% CI 0.350–0.836, *p* = 0.006). For fistula response, the number of setons placed remained a significant independent predictor (adjusted OR 0.515, 95% CI 0.275–0.964, *p* = 0.038).

### 3.5 Stratified analysis by treatment strategy

Patients who underwent definitive surgical intervention demonstrated significantly higher fistula healing rates compared to those managed with medical therapy alone (74.3% vs. 34.4%, *p* < 0.001). Simple fistulas managed with UST alone showed limited healing (25.0%, *n* = 2), precluding definitive conclusions for this subgroup. This benefit was observed across both simple and complex fistula subgroups (Table 5). Biologic-naïve patients achieved higher healing rates than biologic-experienced patients (75.3% vs. 51.2%, *p* = 0.008). Among biologic-experienced patients, those with only one prior biologic (*n* = 45) had better outcomes than those with two or more prior biologics (*n* = 35) (58.6% vs. 40.0%, *p* = 0.089). Simple fistulas (AGA type 0) demonstrated superior healing outcomes compared to complex fistulas (AGA type 1) when combined with surgical intervention (100% vs. 74.3%, *p* = 0.028). However, without surgical intervention, simple fistulas managed with UST alone also showed limited healing (25.0%). To account for the timing of surgical intervention relative to UST initiation, we conducted a time-dependent Cox regression treating surgery as a time-varying covariate. Definitive surgery remained a significant predictor of healing (adjusted HR 4.82, 95% CI 2.91–7.98, *p* < 0.001) after controlling for timing. Excluding patients who underwent definitive surgery before week 16 (*n* = 23), healing rates among surgical patients remained significantly higher than non-surgical patients (71.0% vs. 25.5%, *p* < 0.001). Among patients with fistula response at week 16 (*n* = 121), those who underwent subsequent definitive surgery (*n* = 78) achieved higher healing rates than those managed medically (*n* = 43) (76.9% vs. 34.9%, *p* < 0.001).

**Table 5:** Fistula healing rates by treatment strategy and AGA classification.

Treatment strategy	*n*	Fistula healing rate	95% CI
Simple fistula + UST + Definitive surgery	16	100.00%	79.4–100%
Simple fistula + UST alone	2	25.00%	7.4–92.6%
Complex fistula + UST + Definitive surgery	100	74.30%	64.8–82.2%
Complex fistula + UST alone	43	25.00%	13.2–40.3%
UST + Definitive surgery (all fistulas)	116	74.30%	66.2–81.4%
UST alone (all fistulas)	47	25.50%	14.3–40.0%
Biologic-naïve patients	83	75.30%	65.1–83.6%
Biologic-experienced patients	80	51.20%	40.1–62.3%
Biologic-experienced: 1 prior biologic	45	58.60%	43.2–72.9%
Biologic-experienced: ≥2 prior biologics	35	40.00%	24.6–57.4%

The small sample size of the “Simple fistula + UST alone” group (*n* = 2) limits the generalizability of its healing rate, and conclusions should be interpreted with caution.

### 3.6 Development and validation of a predictive nomogram

**Figure 4:**
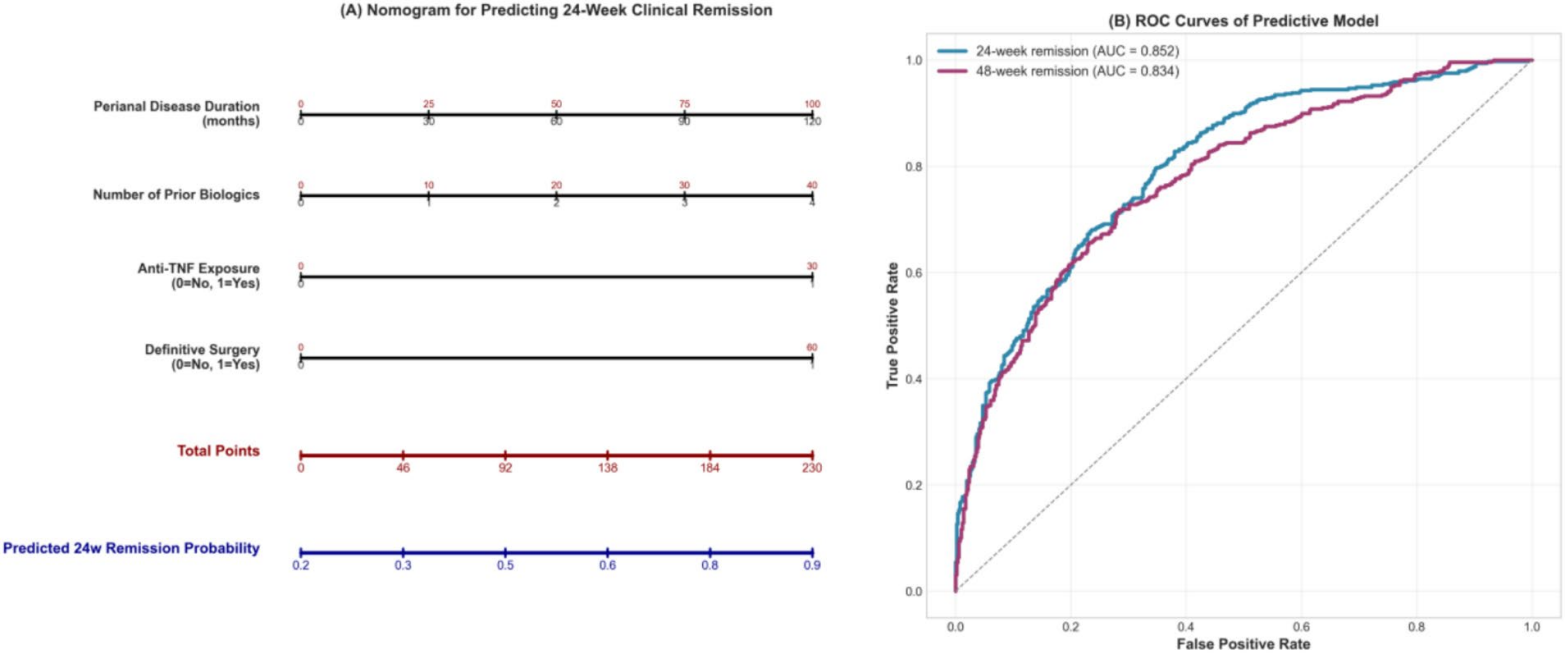
Predictive Nomogram for Clinical Remission in pfCD. **(A)** Nomogram for predicting 24-week clinical remission. Each variable is assigned a point value, and the total points correspond to the predicted probability of 24-week clinical remission. **(B)** ROC curves of the predictive model for 24-week and 48-week clinical remission. The AUC values indicate strong discriminative ability of the model. This nomogram includes definitive surgery as a modifiable predictor and is intended for use after initial UST induction (week 8–16).

To facilitate clinical decision-making, we developed a logistic regression-based nomogram incorporating four independent predictors of clinical remission: perianal disease duration, number of prior biologics, anti-TNF exposure, and definitive surgery (Figure 4A). The model demonstrated good discriminative ability with an area under the receiver operating characteristic curve (AUC) of 0.852 (95% CI 0.784–0.920) for 24-week clinical remission and 0.834 (95% CI 0.762–0.906) for 48-week remission (Figure 4B). Calibration plots showed good agreement between predicted and observed probabilities (Hosmer-Lemeshow test, *p* = 0.42). The nomogram enables individualized risk estimation and may guide treatment intensification decisions. The nomogram exhibited good discriminative ability with an area under the receiver operating characteristic curve (AUC) of 0.852 (95% CI 0.784–0.920) for 24-week remission and 0.834 (95% CI 0.762–0.906) for 48-week remission. Bootstrap internal validation (1,000 iterations) yielded an optimism-corrected AUC of 0.831. Calibration plots showed good agreement between predicted and observed probabilities (Hosmer-Lemeshow test, *p* = 0.42).

### 3.7 Safety and tolerability

UST was generally well-tolerated. Adverse events were reported in 12 patients (7.4%): injection site reactions (*n* = 5), upper respiratory tract infections (*n* = 4), and rash (*n* = 3). All infections were mild (managed outpatient); no Grade ≥3 infections, malignancies, or deaths occurred. One patient discontinued UST due to persistent rash. Concomitant corticosteroids (12.9%) or immunosuppressants (23.3%) at baseline showed no significant signal for increased toxicity (*p* > 0.05 for both), though event numbers were small. Median UST exposure was 15 months. Postoperative complications (wound infection, delayed healing) were recorded in surgical databases but not systematically captured as UST-related adverse events. The adverse event rate of 7.4% reflects UST-specific tolerability in a population screened for acute surgical complications; the exclusion of patients with intestinal perforation or recent major abdominal surgery may underestimate the overall safety burden in unselected pfCD patients.

Among patients achieving fistula healing at any timepoint (*n* = 114), 89.5% (*n* = 102) maintained healing at final follow-up without additional interventions. Eleven patients (9.6%) experienced fistula recurrence after initial healing; 7 of these were successfully managed with UST dose escalation (every-4-week dosing) combined with repeat seton placement, while 4 required transition to alternative biologics (vedolizumab *n* = 3, risankizumab *n* = 1).

The median time to fistula healing was 24 weeks (IQR 16–40 weeks) for patients receiving combined medical-surgical therapy, compared to 48 weeks (IQR 24–72 weeks) for those managed medically alone (*p* = 0.003).

## 4 Discussion

This multicenter retrospective study suggested that UST demonstrates clinically meaningful efficacy for long-term pfCD treatment, with sustained fistula response (88.3%) and clinical remission (70.1%). However, the modest MRI healing rate (14.2%) indicates that clinical closure may overestimate true fistula resolution, and radiological assessment remains essential for evaluating deep tissue healing. Significant improvements were observed in inflammatory biomarkers (CRP, ESR) and hemoglobin levels. Multivariate analysis identified definitive surgery (adjusted OR = 9.613, *p* < 0.001) and fewer prior biologics (adjusted OR = 0.541, *p* = 0.006) as independent predictors of fistula healing. Patients receiving combined UST-surgical therapy achieved substantially higher healing rates than those on medical therapy alone (74.3% vs. 34.4%, *p* < 0.001). A predictive nomogram incorporating perianal disease duration, prior biologics, anti-TNF exposure, and definitive surgery demonstrated good discriminative ability (AUC = 0.852 for 24-week remission). UST was well-tolerated with an adverse event rate of 7.4% and low recurrence rate (9.6%).

The demographic characteristics of our cohort align with established epidemiological patterns of pfCD: male predominance (73.6%), early age at onset (median 26 years), and high prevalence of complex fistulas (89.0%) (14, 15). These baseline features confirm that our study population is representative of real-world pfCD patients, enhancing the generalizability of our findings. Our clinical remission rates differ from prior studies, but direct comparisons should be interpreted cautiously due to differences in outcome definitions, surgical intervention rates (71.2% in our cohort vs. variable/lower rates in prior studies), and follow-up durations. Our results likely reflect combined medical-surgical therapy rather than UST pharmacological efficacy alone. A post-hoc analysis of the UNITI-1/UNITI-2 trials reported 24.7% fistula closure at week 8 and 80% clinical fistula response at week 44 (16), while the BioLAP study—the largest retrospective pfCD cohort to date—observed therapeutic success in 38.5% of UST-treated patients (17). A prospective Dutch study reported a 35.7% clinical fistula remission rate at 24 weeks (18). These studies differ in design and context: UNITI was a controlled trial with standardized follow-up; BioLAP used variable surgical protocols; the Dutch cohort was prospective but surgery-sparing. Our cohort demonstrated 29.6% clinical remission at week 24, 64.2% at week 48, and 70.1% at final follow-up (median 15 months), with fistula response rates of 88.7% at week 24 and 88.3% at final follow-up. These apparent differences likely reflect contextual factors rather than differential drug efficacy: (1) our integrated medical-surgical model with high definitive surgery rates; (2) longer observation windows allowing delayed healing; and (3) inclusion of biologic-naïve patients reflecting regional treatment sequencing. We do not claim superiority of UST in our setting; rather, these data characterize combined multidisciplinary efficacy.

Notably, MRI healing rates in our study remained modest (14.2% at final follow-up), despite higher clinical remission rates. This disparity highlights the limitations of clinical examination alone and underscores the importance of radiological assessment in evaluating deep tissue healing. The observed MRI improvement rate of 30.8% at week 24 suggests that partial radiological response may precede complete clinical remission, supporting the use of MRI as a complementary outcome measure in pfCD trials (19).

Our findings strongly support a multidisciplinary approach to pfCD management. Patients who underwent definitive surgical intervention achieved a significantly higher healing rate than those managed with medical therapy alone (74.3% vs. 34.4%, *p* < 0.001). This benefit was observed across both simple and complex fistula subgroups, with simple fistulas achieving 100% healing when combined with surgery, while complex fistulas still demonstrated substantial benefit (74.3% healing rate). Multivariate analysis identified definitive surgery as the strongest independent predictor of fistula healing (adjusted OR = 9.613, 95% CI 4.047–20.747, *p* < 0.001), with nearly ten-fold increased odds of healing compared to medical therapy alone. This finding aligns with the pathophysiology of pfCD, where persistent sepsis and epithelialized tracts perpetuate inflammation despite immunosuppressive therapy (20–22). Surgical intervention addresses these structural abnormalities, allowing UST to effectively control underlying mucosal and systemic inflammation and prevent recurrence. The marked gap between clinical (70.1%) and MRI (14.2%) healing rates warrants careful interpretation. Clinical examination assesses superficial drainage cessation, whereas MRI evaluates deep tract anatomy and inflammatory activity. Persistent MRI abnormalities in clinically “healed” patients may represent subclinical inflammation with recurrence risk. The high surgical rate (71.2%) achieved anatomical closure but may not allow sufficient time for radiological remodeling. Seton removal upon clinical improvement may also precede MRI resolution. These findings underscore that clinical remission alone may be insufficient to declare true fistula resolution.

The high rate of definitive surgical intervention (71.2%) and its status as the strongest predictor of healing (adjusted OR 9.613) raise an important interpretive issue: the observed outcomes likely reflect combined medical-surgical therapy rather than UST efficacy in isolation. We have addressed this by: (1) reframing the study as evaluation of UST within a multidisciplinary strategy; (2) clarifying that all patients received UST prior to definitive surgery, with surgery typically performed after initial inflammatory control; (3) performing sensitivity analyses excluding early surgical cases; and (4) Presenting stratified outcomes by treatment strategy. These analyses confirm that the superiority of combined therapy is robust. Nevertheless, we caution that our data cannot establish the independent contribution of UST versus surgery to healing. Future randomized studies comparing UST + surgery versus surgery alone or UST alone are needed to disentangle these effects.

The superiority of combined therapy was further evident in MRI outcomes, with higher radiological healing rates in the surgical group. This suggests that surgery not only achieves clinical closure but also facilitates deep tissue healing, potentially reducing long-term recurrence risk. Our data support current guideline recommendations advocating for early surgical consultation in pfCD management and challenge the traditional paradigm of sequential medical-then-surgical therapy (6, 23).

A key finding of our study is the negative impact of prior biologic exposure on UST efficacy. Biologic-naïve patients achieved a 75.3% healing rate compared to 51.2% in biologic-experienced patients (*p* = 0.008), and each additional prior biologic agent reduced the odds of healing by 46% (adjusted OR = 0.541, 95% CI 0.350–0.836, *p* = 0.006). This dose–response relationship suggests that cumulative biologic failure may indicate more refractory disease or the development of treatment resistance mechanisms (24). Among biologic-experienced patients, those with only one prior biologic had better outcomes (58.6%) than those with two or more prior biologics (40.0%), although this difference did not reach statistical significance (*p* = 0.089), likely due to limited sample size. Nevertheless, the trend supports positioning UST earlier in the treatment algorithm, before the development of multi-drug resistance (25). Notably, despite reduced efficacy in biologic-experienced patients, UST still provided meaningful benefit, with a healing rate exceeding 50% even in this refractory population, confirming its value as a salvage therapy after anti-TNF failure (26). The mechanism underlying reduced response with prior biologic exposure may involve irreversible structural damage from prolonged inflammation, alterations in the immunological milieu, or selection of treatment-resistant disease phenotypes (17, 27). These findings have important implications for treatment sequencing in pfCD, suggesting that early introduction of effective biologics like UST, potentially in combination with surgery, may optimize long-term outcomes.

To address the lack of validated predictive tools for pfCD, we developed a logistic regression-based nomogram incorporating four independent predictors: perianal disease duration, number of prior biologics, anti-TNF exposure, and definitive surgery. The model demonstrated good discriminative ability with AUC values of 0.852 for 24-week and 0.834 for 48-week clinical remission, indicating robust predictive performance. This nomogram addresses a critical unmet need in pfCD management by enabling individualized risk stratification (28). Clinicians can estimate a patient’s probability of response based on readily available clinical variables, identify those likely to benefit from early surgical intervention or dose escalation, and optimize resource allocation. The inclusion of definitive surgery as a modifiable factor in the nomogram is particularly valuable, as it allows clinicians to simulate how surgical intervention might improve outcomes for individual patients. This supports shared decision-making and reinforces the importance of multidisciplinary care in pfCD (29).

UST was well-tolerated in our cohort, with an adverse event rate of 7.4% and no severe adverse events, consistent with its established safety profile in luminal CD (19, 30). The low fistula recurrence rate (9.6%) at final follow-up further supports the durability of UST efficacy. Among patients who experienced relapse, the majority (7/11) were successfully managed with dose escalation to every-4-week dosing combined with repeat seton placement, avoiding the need for treatment discontinuation or switching. These findings align with previous reports demonstrating the effectiveness of UST dose intensification in recapturing response (31). The ability to escalate dosing without significant safety concerns provides a valuable therapeutic option for patients with suboptimal response, potentially delaying or avoiding the need for alternative biologics.

Our study has several limitations that warrant consideration. First, the retrospective design introduces inherent selection bias and residual confounding. The cohort is enriched with complex fistulas (89%) and prior surgical interventions (85.9%), reflecting referral patterns of tertiary surgical centers where refractory patients are concentrated. The exclusion of 23 screened patients (12.4%) for insufficient follow-up or incomplete records could theoretically bias toward responders; however, baseline characteristics were comparable between included and excluded patients, and week 24 retention was high (99.4%), minimizing differential attrition. The absence of a placebo or active comparator group limits causal inference and direct comparisons with other biologics. Second, the inclusion of definitive surgery as a predictor introduces treatment paradox bias, as surgery is a treatment decision rather than a baseline characteristic. The nomogram predicts healing under combined medical-surgical therapy, not UST efficacy in isolation. The small sample size of the “simple fistula + UST alone” subgroup (*n* = 2) precludes definitive conclusions about outcomes in this specific population. Third, while our median follow-up of 15 months is longer than many previous studies, it may not capture ultra-long-term outcomes beyond 2–3 years, including late recurrences or cumulative safety events. Fourth, MRI data were not available for all patients, and clinical remission was primarily assessed by physical examination, which may be less sensitive than radiological assessment for detecting subclinical disease activity. The discrepancy between clinical (70.1%) and MRI (14.2%) healing rates suggests that clinical examination may overestimate true healing, although the clinical significance of persistent MRI abnormalities in asymptomatic patients remains debated (30). Whether clinical remission without MRI confirmation predicts sustained remission remains uncertain and requires longer-term follow-up. Finally, as a single-region multicenter study conducted in Chinese patients, the results may not be fully generalizable to other ethnic populations with different genetic backgrounds, disease phenotypes, or healthcare systems. Prospective validation in diverse populations is needed to confirm the generalizability of our findings and the predictive nomogram. The high rate of combined surgical intervention (71.2%) limits our ability to attribute outcomes solely to UST. Our results reflect real-world multidisciplinary practice but cannot establish the independent efficacy of UST in the absence of surgery. The lack of a surgery-only or UST-only comparator group precludes causal inference about the relative contributions of each modality. The apparent decline in fistula response at week 48 (46.7%, *n* = 75) reflects right censoring—patients enrolled after January 2022 had not reached 48 weeks at the December 2023 data cutoff—rather than selective attrition. This precludes formal longitudinal modeling; future prospective studies should employ such approaches. The 7.4% adverse event rate may underestimate mild, undocumented events due to retrospective data collection; exposure-adjusted stratification by combination therapy was limited by small event numbers. Our safety findings should be interpreted with caution due to the exclusion of patients with intestinal perforation during UST therapy and major abdominal surgery within 3 months before or during treatment. These exclusions were necessary to isolate the therapeutic effect of UST on fistula healing from confounding surgical morbidity, but they likely underestimate the true incidence of serious adverse events in the broader pfCD population. Intestinal perforation and postoperative complications are well-documented complications of Crohn’s disease and its surgical management, and their exclusion from our cohort means our safety profile may not capture the full range of clinically significant adverse outcomes. Future studies without such exclusions are needed to establish the comprehensive safety profile of UST in real-world pfCD practice.

Our findings have several important clinical implications. First, they support the positioning of UST as an effective therapy for pfCD, with particular emphasis on its use earlier in the treatment sequence before multiple biologic failures. Second, they underscore the critical importance of combined medical-surgical therapy, challenging the traditional sequential approach and supporting simultaneous or early surgical intervention. Third, the validated nomogram provides a practical tool for individualized treatment planning and patient counseling.

Future research should focus on prospective validation of our findings in randomized controlled trials, ideally comparing UST with other advanced therapies (vedolizumab, risankizumab) in biologic-experienced patients. Longer-term studies are needed to assess durability of response beyond 2–3 years and to determine whether deep radiological healing predicts sustained clinical remission and reduced surgery rates. Additionally, biomarker-driven studies could identify molecular signatures predicting response to UST, potentially enabling precision medicine approaches in pfCD.

## 5 Conclusion

UST demonstrates clinically meaningful efficacy within a combined multidisciplinary strategy for pfCD, with high clinical response rates. However, the limited MRI healing rate (14.2%) indicates that clinical closure may not reflect true deep tissue resolution. Definitive surgery is the strongest predictor of fistula healing, and combined UST-surgical therapy yields substantially superior outcomes compared to medical therapy alone. The predictive nomogram facilitates individualized treatment decision-making. Definitive surgery and fewer prior biologic exposures are independent predictors of favorable outcomes, and combined UST-surgical therapy yields superior results compared to medical monotherapy. The developed predictive nomogram provides a practical tool for individualized treatment decision-making. These findings strengthen the evidence for UST as a key therapeutic option in pfCD and highlight the importance of a multidisciplinary, individualized approach to optimize patient outcomes.

## Data Availability

The original contributions presented in the study are included in the article/supplementary material, further inquiries can be directed to the corresponding author.
